# Combined Treatment of Large Fusiform A2 Aneurysm with End-to-Side Extended Superficial Temporal Artery–A3 Bypass Using Contralateral Superficial Temporal Artery Interposition Graft and Endovascular Aneurysm Trapping: A Case Report and Literature Review

**DOI:** 10.3390/jcm14092927

**Published:** 2025-04-24

**Authors:** You-Sub Kim, Jae-Woong Kim, Woong-Beom Kim, Byung-Hyun Baek, Woong Yoon, Tae-Sun Kim, Sung-Pil Joo

**Affiliations:** 1 Department of Neurosurgery, Chonnam National University Hospital and Medical School, Gwangju 61469, Republic of Korea; neohoyo@naver.com (J.-W.K.); kyler-kwb@hanmail.net (W.-B.K.); taesun1963@yahoo.co.kr (T.-S.K.); 2 Department of Radiology, Chonnam National University Hospital and Medical School, Gwangju 61469, Republic of Korea; qorqod10@gmail.com (B.-H.B.); radyoon@chonnam.ac.kr (W.Y.)

**Keywords:** A2, fusiform aneurysm, anterior cerebral artery, bypass, endovascular treatment

## Abstract

**Background:** A2 fusiform aneurysms present certain management difficulties with conventional microsurgical or endovascular approaches due to the circumferential morphology, deep location within the interhemispheric fissure, and narrow surgical corridor. **Methods:** We present a case of a large (>10 cm) fusiform aneurysm in the right A2 segment treated with a combined method consisting of an extended superficial temporal artery to A3 bypass using a contralateral superficial temporal artery interposition graft and subsequent endovascular trapping of the aneurysm. To treat the aneurysm, endovascular trapping following revascularization was planned. During surgery, as the left A3 segment was not available, a superficial temporal artery to A3 bypass was performed. The right frontal branch (donor) was extended with the left frontal branch as a free interposition graft (end-to-end anastomosis) and then anastomosed with the right A3 segment (end-to-side anastomosis). At 6 days after surgery, after confirming the good patency of the bypass graft, endovascular aneurysm trapping was performed. **Results:** At 8 days after surgery, the patient was discharged without any neurologic deficits. Follow-up digital subtraction angiography at 12 months after surgery showed the good patency of the bypass graft with complete occlusion of the aneurysm. **Conclusions:** Our case demonstrates the feasibility and effectiveness of a combined microsurgical-endovascular approach as a management strategy for deeply located A2 fusiform aneurysm. When in situ bypass is not possible, an extended superficial temporal artery donor may be considered.

## 1. Introduction

Fusiform aneurysms are specifically characterized by circumferential dilation of vessels without a discrete neck and are associated with a high rate of rupture [1,2]. While fusiform aneurysms account for only 3–13% of all cerebral aneurysms, their prevalence is considerable in the posterior circulation [3,4]. The fusiform A2 aneurysm in the anterior cerebral artery (ACA) is particularly rare and presents unique challenges for treatment due to the deep location within the interhemispheric fissure, narrow surgical corridor and involvement of branching vessels [2,5]. Several surgical techniques have been introduced to treat fusiform A2 aneurysms, including simple clipping, proximal occlusion, and trapping with or without bypass [2,6,7]. Recently, the evolution of endovascular treatment such as flow-diverting and stent-assisted techniques have similarly expanded treatment options for fusiform A2 aneurysms, with long-term studies showing favorable outcomes [8,9]. Despite emerging multidisciplinary approaches, each treatment is associated with a significant degree of risk due to its complexity, and the critical nature of the A2 segment of the ACA underscores a continued need for individualized treatment planning based on aneurysm characteristics. Here, we present a rare case of a large fusiform A2 aneurysm successfully treated with a combined method consisting of an extended superficial temporal artery (STA) to A3 bypass and endovascular occlusion of the aneurysm and parent artery.

## 2. Case Presentation

**Figure 1:**
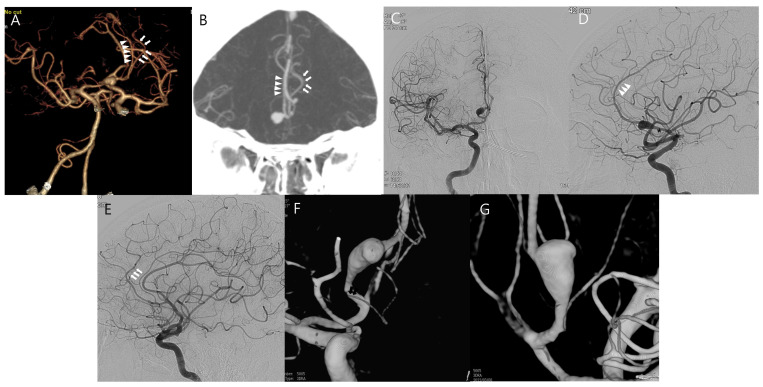
Brain images from a 65-year-old male who presented with sustained headache. (**A**,**B**) Initial computed tomography angiography shows a fusiform aneurysm at the A2 segment of the anterior cerebral artery. (**C**,**D**) Right antero-posterior and lateral internal carotid artery angiogram shows fusiform aneurysm at A2. Note that the proximal portion of the aneurysm (asterisk) is deep and anteriorly deformed parallel to the aneurysm. (**E**) The caliber of left A3 (white arrows) is similar to right A3 (white arrowheads) of the bypass site; however, (**A**,**B**) there exists significant distance between them. (**F**,**G**) The aneurysm is located just distally to the frontopolar artery (black arrows) with bleb and a maximal size of 10 cm.

**Figure 2:**
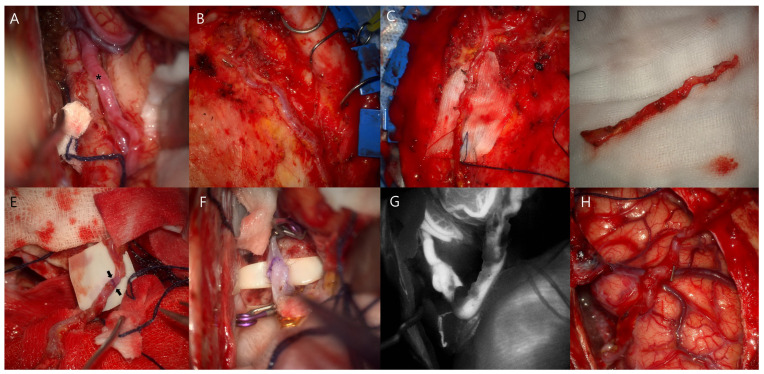
Intraoperative findings. (**A**) Following bifrontal craniotomy and interhemispheric dissection, the right callosomarginal artery (A3 segment, asterisk) was exposed. The left callosomarginal artery was not available for in situ A3–A3 bypass. (**B**–**D**) The right and left frontal branches of the superficial temporal artery (STA) were dissected to extend the right frontal branch (donor) using the left frontal branch as a free interposition graft. (**E**,**F**) After end-to-end anastomosis between the frontal branches of the STA (black arrows), (**F**) end-to-side anastomosis was performed between the extended STA graft and right callosomarginal artery. (**G**,**H**) The bypass graft showed good patency.

**Figure 3:**
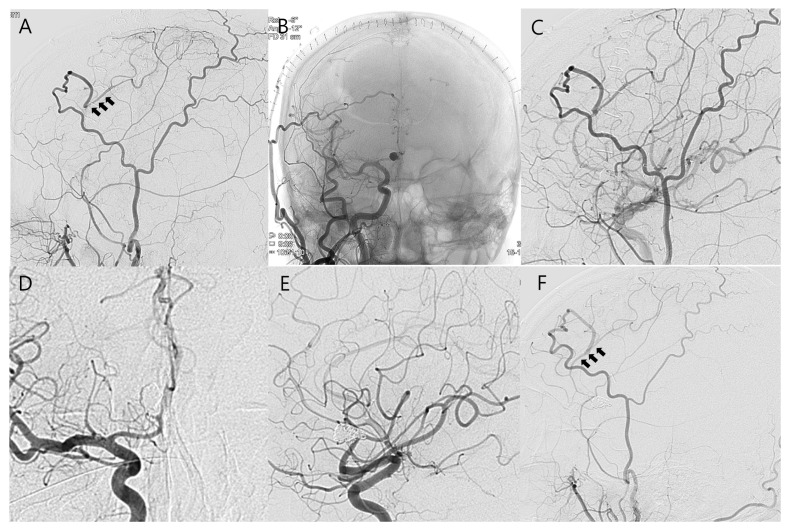
Endovascular treatment and imaging follow-up. (**A**) Postoperative right external carotid artery angiography reveals the good patency (black arrows) of the STA–A3 bypass graft. (**B**,**C**) Endovascular coil occlusion of the aneurysm and parent artery was performed. (**D**–**F**) Follow-up angiography at 12 months after surgery showed the good patency of the bypass graft (black arrows) with no evidence of recurrence.

A 65-year-old man was admitted with sustained headache. Computed tomography angiography (CTA) revealed fusiform aneurysmal dilatation at the A2 segment of the right ACA. (Figure 1A) Digital subtraction angiography (DSA) demonstrated a large fusiform aneurysm (maximal length > 10 cm) with bleb formation at the right A2 segment of the ACA, just distally to the frontopolar artery. (Figure 1C–G) We initially decided to perform revascularization and trapping for complete cure to prevent future rupture or ischemic complications considering that simple clipping or endovascular treatment alone, including flow diversion, is not the first-line treatment option for fusiform aneurysms. Given the course and location of the fusiform aneurysms of the right A2 segment with a deep proximal portion (Figure 1D), it would be invasive and require a long time to perform revascularization and trapping of the aneurysm simultaneously during surgery due to the large bone flap and wide interhemispheric dissection to expose not only the space for bypass but the proximal portion of the aneurysm. Therefore, we planned for combined treatment comprising initial revascularization to maintain the distal arterial supply, followed by endovascular occlusion of the aneurysm and parent artery. Following bifrontal craniotomy, the interhemispheric fissure was dissected. The right A3 segment was sufficiently exposed to perform bypass; however, in situ A3–A3 bypass was not possible because the left A3 segment of the ACA was not visible. (Figure 2A) Actually, the caliber of left A3 is similar to right A3; however, both A3s are not in close proximity in preoperative CTA and DSA. (Figure 1A,B,D,E) We decided to perform the bypass using the STA instead of in situ A3–A3 bypass. The frontal branches of the both STAs were dissected. (Figure 2B,C) As the right frontal branch of the STA alone was too short to reach the right A3 segment, it was extended with the left frontal branch of the STA as a free interposition graft (end-to-end anastomosis, Figure 2D,E) and then anastomosed with the right A3 segment of the ACA (end-to-side anastomosis, Figure 2F). Intraoperative angiography and doppler confirmed the good patency of the bypass graft. (Figure 2G,H) After 6 days, DSA was performed, which confirmed the patent distal flow through the bypass graft, and the aneurysm and parent artery were completely occluded with 13 detachable coils. (Figure 3A–C) At 8 days after surgery, the patient was discharged without any neurologic deficits. Follow-up DSA at 12 months after surgery showed the good patency of the bypass graft with complete occlusion of the aneurysm (Figure 3D–F).

## 3. Discussion

Fusiform aneurysms of the ACA, particularly at the A2 segment, represent an exceedingly rare entity [1,5]. Consequently, there are inherent challenges in establishing standardized treatment protocols, as most neurosurgeons encounter these cases infrequently. The complexity of A2 fusiform aneurysms, characterized by circumferential dilation without a discrete neck, deep location, critical perforating arteries, and the narrow working corridor of the interhemispheric fissure, makes them particularly challenging to treat using conventional approaches. In this context, the selection of appropriate treatment necessitates a comprehensive evaluation of patient-specific factors such as collateral circulation and comorbidities as well as aneurysm characteristics, including size, location, and involvement of perforating arteries [10]. The critical nature of the distal ACA territory increases the risk of treatment decisions, as treatment-related complications can result in significant neurological deficits. As a result, various surgical and endovascular treatments have been performed within limited cases of A2 fusiform aneurysm, as described in Table 1.

Recent advancements in endovascular treatment for treating fusiform distal ACA aneurysms, including A2 aneurysms, have focused on a reconstructive method using flow-diverting stents and stent-assisted techniques, which represent a paradigm shift from conventional endovascular approaches. Dabus et al. obtained promising results using pipeline flow diversion for complex ACA aneurysms, reporting complete or near-complete occlusion in 75% of cases at the mid-term follow-up [8]. The evolution of stent-assisted techniques has similarly expanded treatment options, with long-term studies showing favorable outcomes for stent reconstruction of fusiform aneurysms [9]. However, besides the significant incidence of recurrence, postoperative ischemic complications and in-stent stenosis, most lesions reported were internal carotid artery or posterior circulation, and as a result, the efficacy was even more uncertain for c distal ACA aneurysms following flow-diverting stents or stent-assisted techniques [4,13]. Moreover, although endovascular techniques are advantageous due to reduced invasiveness, concerns remain regarding the possibility of incomplete aneurysm occlusion and the necessity for long-term dual antiplatelet therapy, particularly in cases where perforator vessels are incorporated into the aneurysmal segment [17,18].

Microsurgical management of an A2 fusiform aneurysm typically involves revascularization procedures to maintain distal flow while excluding the aneurysm from circulation. Until recently, A3–A3 in situ bypass with aneurysm trapping has been increasingly proposed as an effective method for most distal ACA aneurysms. Several case series and reports have indicated successful treatment of large fusiform distal ACA aneurysms using this technique, highlighting its potential for complete aneurysm obliteration while preserving distal flow [2,7,14,16]. The A3–A3 bypass technique offers several advantages, including the use of similar-caliber vessels for anastomosis, a short graft distance, and the ability to perform the procedure within a single surgical field. 

However, this approach is not always available and often limited by the involvement of branch vessels or inadequate recipient vessels, which are not in close proximity or are not similar in caliber with recipient vessels [19]. Besides, simultaneous A3–A3 bypass followed by aneurysm trapping presents considerable technical challenges, including the need for extensive interhemispheric dissection, the risk of venous injury, and the excessive bilateral frontal lobe retraction [10]. These factors can contribute to longer operative times and increased risk of procedure-related morbidity, particularly in cases where the proximal portion of an aneurysm is deeply located or difficult to access. Our treatment addresses several issues in the current management approaches for A2 fusiform aneurysms. First, given the course and location of fusiform aneurysms of the right A2 segment with a deep proximal portion, we recognized that conventional approaches involving simultaneous revascularization and trapping would require extensive bone removal and interhemispheric dissection. This would substantially increase surgical invasiveness and operative time, potentially elevating the risk of complications. Our hybrid approach strategically separated these components—first establishing revascularization to secure distal perfusion, followed by endovascular occlusion of the aneurysm and parent artery. Second, when interhemispheric dissection revealed that in situ A3–A3 bypass was not feasible due to the inadequate donor or recipient vessels, extended STA can be an useful option for revascularization. Our strategy of extending the right frontal branch of the STA with the left frontal branch as a free interposition graft is the first report in treating A2 fusiform aneurysm and represents a flexible approach for overcoming anatomical or technical limitations. Third, the staged approach also allows for confirmation of bypass patency before proceeding with parent vessel sacrifice, providing an additional safety margin not available with single-stage procedures.

## 4. Conclusions

A2 fusiform aneurysms represent a rare and challenging neurovascular pathology that continues to reach the limits of both endovascular and microsurgical techniques without established consensus for standard treatment. Our hybrid approach, comprising revascularization through an extended STA–A3 bypass followed by endovascular parent vessel occlusion, can provide another available treatment option for A2 fusiform aneurysms in which one-step treatment or in situ bypass is not feasible due to their anatomical problems.

## Figures and Tables

**Table 1:** Summary of the literature on A2 fusiform aneurysms.

No	Author (Year)	Age/Sex	Clinical Presentation	Treatment	Complications	Follow-Up
1	Anson et al. (1996) [1]	64/F	Vision loss	Distal occlusion, A2–A2 side-to-side bypass	None	3.2 years
2	Anson et al. (1996) [1]	34/F	Seizure	Aneurysm resection	None	3.3 years
3	Fuentes et al. (2004) [11]	24/F	Vision loss	Endovascular parent artery occlusion, aneurysm excision	None	N/A
4	Park et al. (2008) [12]	N/A	N/A	N/A	N/A	N/A
5	Devulapalli et al. (2013) [13]	50/F	Subarachnoid hemorrhage	Stent-assisted coiling	Rebleeding	N/A
6	Chen et al. (2014) [2]	46/M	Subarachnoid hemorrhage	Clip-wrapping	N/A	N/A
7	Chen et al. (2014) [2]	51/M	Bitemporal hemianopsia	A3–A3 side-to-side bypass, aneurysm trapping (surgical and endovascular)	N/A	N/A
8	Chen et al. (2014) [2]	51/F	Incidental	A3–A3 side-to-side bypass, aneurysm trapping	N/A	N/A
9	Chen et al. (2014) [2]	18/F	Incidental	Frontopolar–A2 bypass, aneurysm resection	N/A	N/A
10	Alurkar et al. (2014) [6]	32/F	Subarachnoid hemorrhage	Endovascular parent artery occlusion	Transient lower limb weakness	1 month
11, 12	Dabus et al. (2014) [8]	N/A	N/A	Pipeline flow diversion	None	N/A
13	Safavi-Abbasi et al. (2017) [14]	N/A	N/A	A3–A3 side-to-side bypass, aneurysm trapping	N/A	N/A
14	Safavi-Abbasi et al. (2017) [14]	N/A	N/A	A3–A3 side-to-side bypass, aneurysm trapping	N/A	N/A
15	Hendricks et al. (2019) [15]	N/A	Incidental	Clip-wrapping	N/A	N/A
16	Hendricks et al. (2019) [15]	31/F	Subarachnoid hemorrhage	A3–A3 side-to-side bypass, aneurysm trapping	None	N/A
17	Gomez-Vega et al. (2024) [16]	52/M	Subarachnoid hemorrhage	A3–A3 side-to-side bypass, aneurysm trapping	None	N/A
18	Present case	65/M	Incidental	Extended STA–A3 bypass, endovascular aneurysm trapping	None	1 year

Abbreviations: N/A, non-applicable; STA, superficial temporal artery.

## Data Availability

The data that support the findings of this study are available from the corresponding author upon reasonable request.

## Abbreviations

The following abbreviations are used in this manuscript:

ACA	Anterior cerebral artery
STA	Superficial temporal artery
DSA	Digital subtraction angiography
